# 

**Published:** 1899-02-25

**Authors:** 


**Notices of Births, Deaths, and Marriages** are inserted in this column at a charge of 2s. 6d. for an announcement not exceeding four lines.

### NOTICE TO CORRESPONDENTS.

All MS., Letters, Books for Review, and other matters intended for the Editor should be addressed The Editor, "The Hospital," The Hospital Building, 28 & 29, Southampton Street, Strand, W.C.
The Editor cannot undertake to return rejected MS., even when accompanied by stamped directed envelope.

**Notice to Advertisers and Subscribers.**
All Advertisements, Orders for Copies of The Hospital, and Business Communications should be addressed to **THE MANAGER (Not to the Editor),** The Hospital, The Hospital Building, 28 & 29, Southampton Street, Strand, London, W.C.
The Cost of Subscription, post free, is as follows:—Per week, 2½d.; per quarter, 2s. 8d.; per half-year, 5s. 3d.; per annum, 10s. 6d. Foreign:—Per annum, 15s. 2d., payable, in all cases, in advance.

**NOTICE.—Vol. XXIV. of "The Hospital" (April, 1898, to September, 1898), in handsome cloth binding, is now on sale at the Office of this Journal, price 7s. 6d. Cases for binding the Numbers contained in this Volume 1s. 6d. Index to Vol. XXIV., 2½d., post free.**

**The Monthly Part for March will be ready March 1st. Price 6d., by post 8d.**

Crown 8vo., Paper Covers, 1s.,

SOME HEALTH ASPECTS OF EDUCATION.

By PERCY G. LEWIS, M.D., M.R.C.S., L.S.A.,

Author of "Nursing: Its Theory and Practice."

"A decidedly practical pamphlet, which has for its object the direction of attention to the possible sanitary defects of a child, and the principles to be observed in repairing them."—*School Guardian.*

London: The Scientific Press, Ltd., 28 & 29, Southampton Street, Strand, W.C.

